# Radiologically Occult Bilateral Androgen-Secreting Sex Cord-Stromal Tumors in a Postmenopausal Woman

**DOI:** 10.7759/cureus.116322

**Published:** 2026-09-15

**Authors:** Rebecca Furstenau, Danny Koh

**Affiliations:** 1 Obstetrics and Gynecology, Midwestern University Chicago College of Osteopathic Medicine, Downers Grove, USA; 2 Obstetrics and Gynecology, Northwestern Medicine, Chicago, USA

**Keywords:** androgen-secreting ovarian tumor, female hyperandrogenism, hormone-secreting ovarian neoplasm, leydig cell tumor, ovarian hyperthecosis, ovarian sex cord-stromal tumor, rare ovarian tumor, sertoli-leydig cell tumour, sex cord-stromal tumor

## Abstract

We present the case of a 66-year-old G3P3 postmenopausal woman with hyperandrogenism who presented with hirsutism and androgenetic alopecia and was subsequently diagnosed with bilateral sex cord-stromal tumors (SCSTs). These rare androgen-secreting ovarian neoplasms are typically unilateral and can be difficult to identify with conventional imaging. Despite negative imaging studies, persistent biochemical hyperandrogenism prompted bilateral salpingo-oophorectomy, which revealed bilateral androgen-secreting SCSTs. This case highlights the importance of maintaining a high index of suspicion for occult ovarian neoplasms in postmenopausal women with unexplained hyperandrogenism despite unrevealing imaging.

## Introduction

Sex cord-stromal tumors (SCSTs) are rare ovarian neoplasms, accounting for approximately 8% of all ovarian tumors [1]. These tumors arise from the sex cords, stromal components, or both [1]. Sertoli-Leydig cell tumors are rare mixed-cell neoplasms accounting for less than 0.2% of all ovarian tumors [2,3]. They typically present in women aged 20 to 30 years and are predominantly unilateral [1,3]. Bilateral SCSTs are extremely rare, with only a few cases reported in the literature [4,5]. Clinical manifestations are often related to androgen excess and may include amenorrhea, hirsutism, acne, male-pattern hair loss, and, in some cases, virilization [3].

The diagnosis of these tumors can be challenging, especially in postmenopausal women. A degree of hyperandrogenism may occur after menopause because ovarian estrogen declines more rapidly than androgen production [6]. Consequently, androgen-related symptoms may be attributed to the postmenopausal state, potentially delaying diagnostic evaluation [6]. Additionally, when a workup reveals elevated androgen levels, identifying the source can be difficult. Due to ovarian atrophy, visualization of the ovaries on imaging may be limited, and small androgen-producing ovarian tumors may remain occult [6,7]. Because imaging and hormonal evaluation may fail to identify a definitive source of androgen excess, diagnosis and treatment can be delayed [6,7].

This case describes the unusual occurrence of synchronous bilateral, radiologically occult androgen-secreting SCSTs in a postmenopausal woman. Despite biochemical evidence suggesting an ovarian source of androgen excess, repeated imaging failed to identify an ovarian lesion, and the tumors were diagnosed only after surgical pathology. The presence of two small, histologically distinct tumors in grossly unremarkable ovaries highlights the limitations of imaging in evaluating postmenopausal hyperandrogenism and the importance of maintaining clinical suspicion for an occult ovarian source.

## Case presentation

A 66-year-old G3P3 postmenopausal woman was referred to endocrinology for evaluation of elevated serum testosterone. She reported six months of hair thinning and the recent onset of coarse facial hair. Her past medical history included bilateral ductal carcinoma in situ with recurrence, treated two decades earlier with bilateral mastectomy followed by five years of tamoxifen therapy.

Initial laboratory results revealed a markedly elevated total testosterone of 133 ng/dL and an elevated free testosterone of 19 pg/mL, as seen in Table 1. Dehydroepiandrosterone sulfate (DHEAS) was within the reference range, making an adrenal source less likely and increasing suspicion for an ovarian source.

**Table 1:** Patient laboratory values

Lab	Result	Reference range
Total testosterone (ng/dL)	133	6-86
Free testosterone (pg/mL)	19	0.2-2.0
DHEAS (μg/dL)	150	30-260

A contrast-enhanced CT scan of the abdomen and pelvis (Figure 1) showed no identifiable ovarian masses. Transvaginal ultrasound failed to visualize the ovaries but revealed a thickened endometrial stripe. Due to her history of tamoxifen use, which increases the risk of endometrial pathology, an endometrial biopsy was performed and revealed a benign polyp.

**Figure 1:**
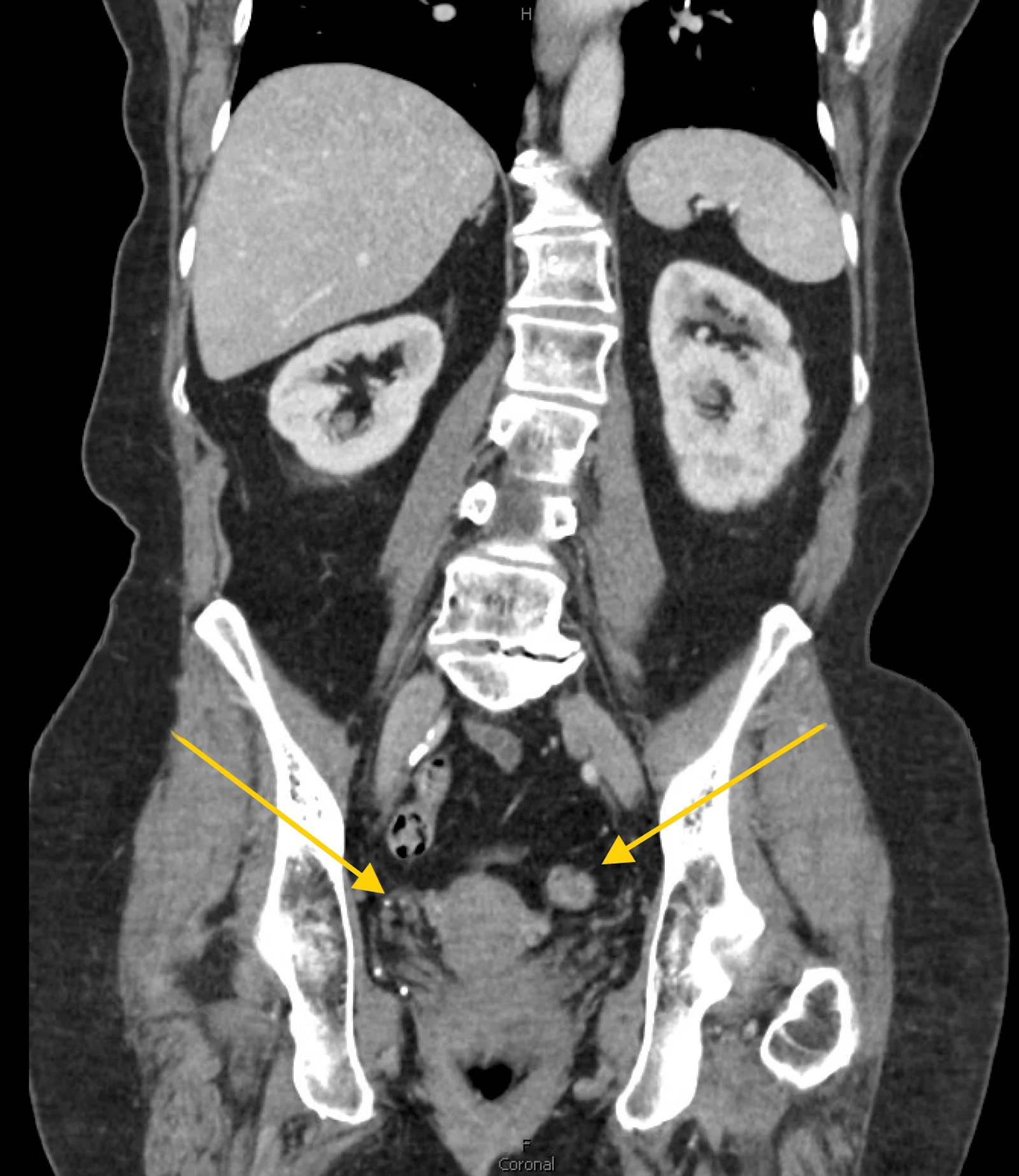
Contrast-enhanced CT of the abdomen and pelvis demonstrating no identifiable adnexal mass Arrows indicate the locations of the bilateral ovaries. No discrete ovarian or adnexal mass is identified despite biochemical evidence of hyperandrogenism. CT: computed tomography

Repeat ultrasonography failed to visualize the ovaries, and the endometrium remained thickened at 7 mm, as seen in Figure 2. Despite negative imaging, the persistent elevations in total and free testosterone levels with a DHEAS level within the reference range continued to raise concern for an occult ovarian source of androgen excess. Given these findings, gynecologic oncology recommended total hysterectomy and bilateral salpingo-oophorectomy for definitive diagnosis and treatment.

**Figure 2:**
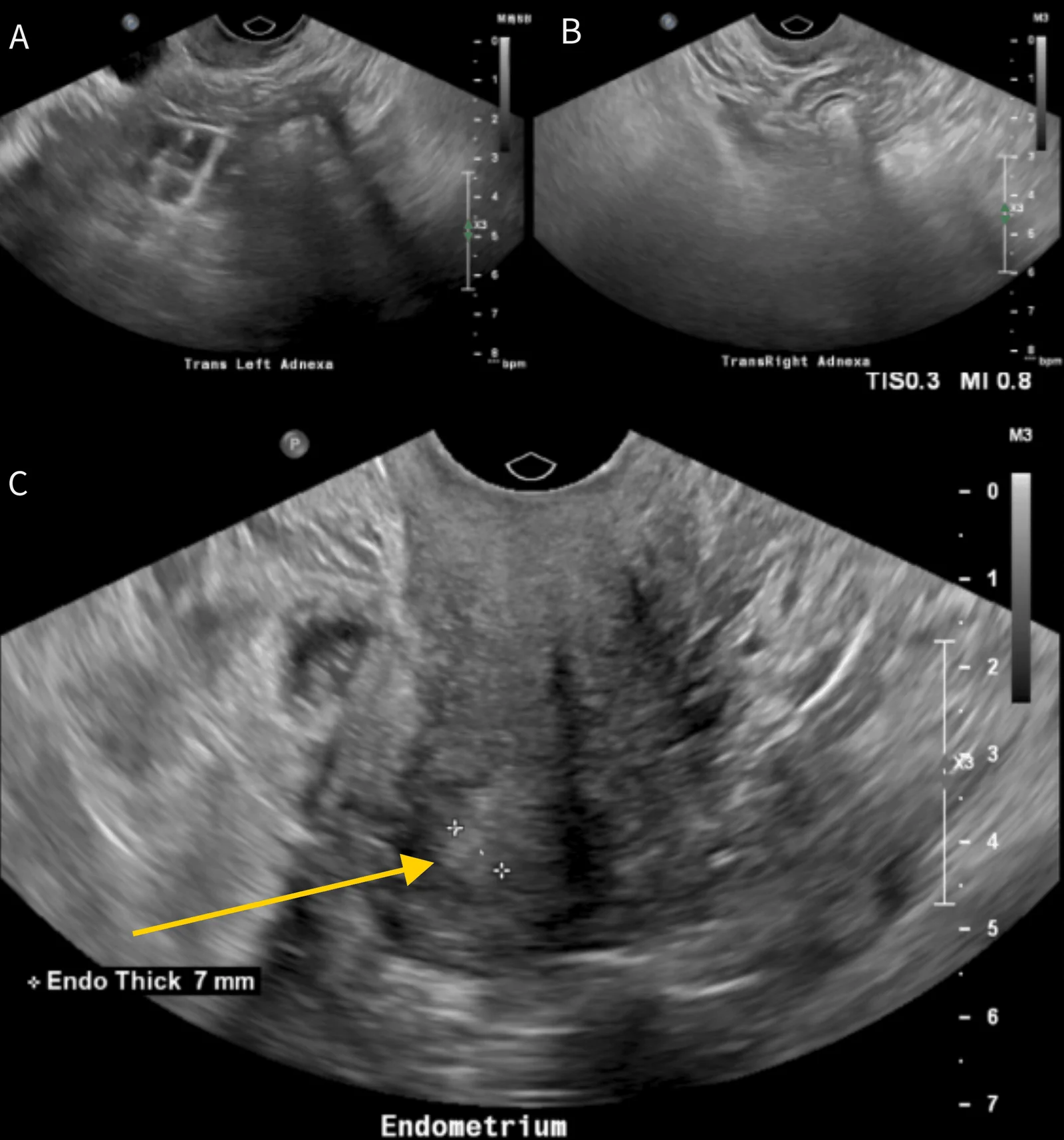
Transvaginal ultrasound demonstrating nonvisualization of the adnexa and a mildly thickened endometrium Transvaginal ultrasound examination. (A) Left adnexal view without identification of an ovary. (B) Right adnexal view without identification of an ovary. (C) Endometrial stripe measuring 7 mm (arrow). No discrete adnexal mass was visualized.

Robotic-assisted laparoscopic surgery was performed. Intraoperatively, the ovaries appeared grossly unremarkable, as seen in Figure 3. However, histopathologic analysis revealed a 0.5-cm Sertoli-Leydig cell tumor in the right ovary and a 0.6-cm well-differentiated (Grade 1) pure Leydig cell tumor in the left ovary. Immunohistochemical staining demonstrated positivity for calretinin and inhibin in both tumor components. The Sertoli cell component additionally showed positivity for cytokeratin AE1/AE3 and weak WT1 expression, while estrogen receptor and PAX8 expression were not significant. These immunohistochemical findings supported the diagnosis of bilateral SCSTs. The patient was clinically staged as FIGO Stage IB. She remains under the care of gynecologic oncology with observational management, including serial inhibin measurements and planned repeat CT imaging.

**Figure 3:**
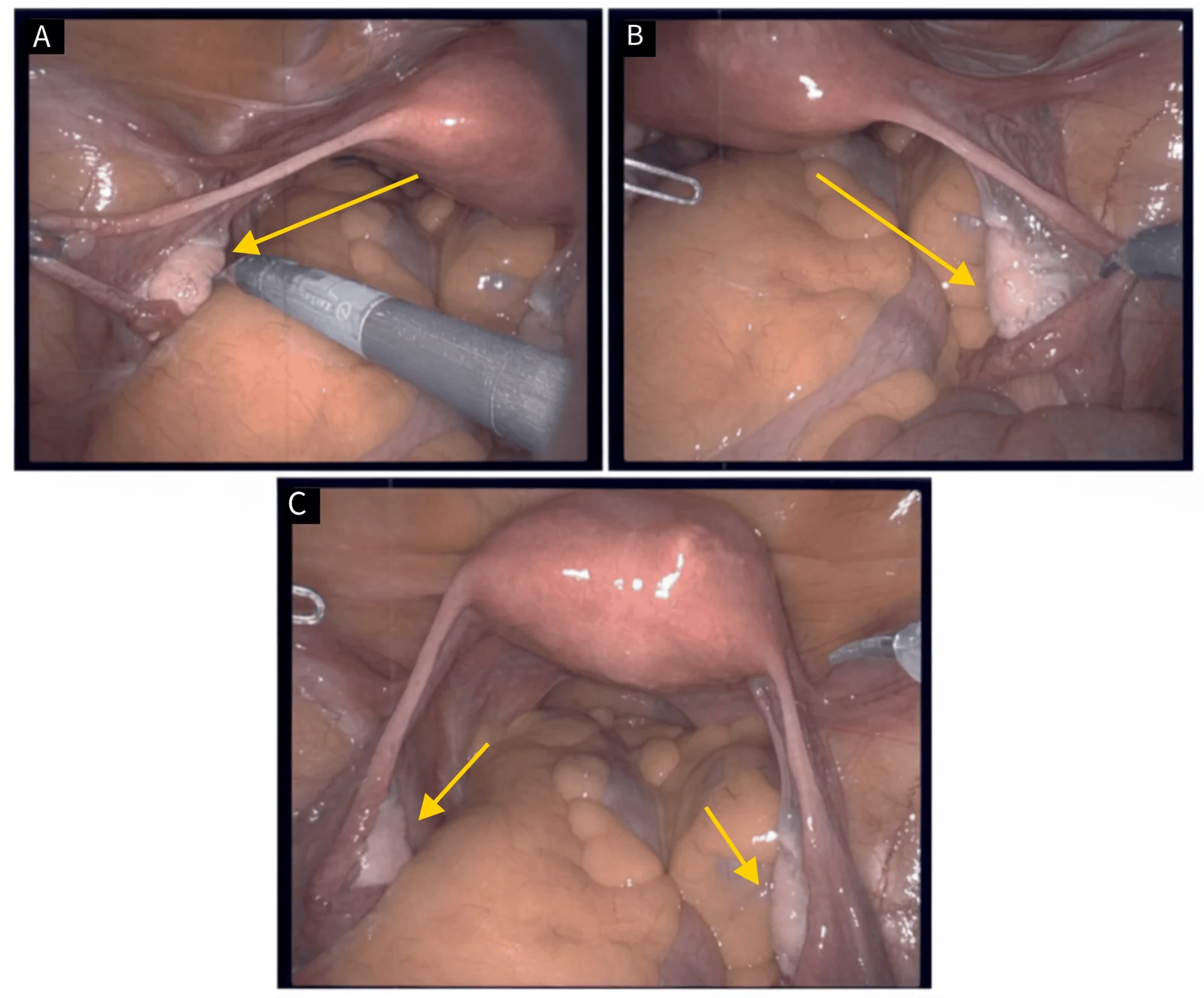
Laparoscopic appearance of grossly normal bilateral ovaries Intraoperative laparoscopic views of the bilateral adnexa. (A) Grossly normal-appearing left ovary (arrow). (B) Grossly normal-appearing right ovary (arrow). (C) Bilateral ovaries in situ (arrows) demonstrating no gross evidence of malignancy despite final pathology revealing bilateral SCSTs. SCSTs: sex cord-stromal tumors

## Discussion

SCSTs are rare ovarian neoplasms that account for approximately 5-8% of all ovarian tumors, while androgen-secreting Sertoli-Leydig cell tumors comprise less than 0.2% of ovarian neoplasms [1-3]. These tumors most commonly occur in women of reproductive age and are typically unilateral [1,3]. In contrast, our patient was postmenopausal and had bilateral androgen-secreting tumors that were not identified on preoperative imaging, making this an exceptionally uncommon presentation [4,5].

The evaluation of hyperandrogenism in postmenopausal women can be particularly challenging. Although mild androgen excess may occur after menopause because of the disproportionate decline in estrogen relative to androgen production, rapidly progressive hirsutism, androgenic alopecia, or virilization should prompt evaluation for an androgen-secreting neoplasm [6]. Initial laboratory testing can help distinguish an ovarian from an adrenal source of androgen excess [6,7]. In this patient, markedly elevated testosterone with a normal DHEAS level strongly suggested an ovarian etiology. Once biochemical testing suggests the source, transvaginal ultrasonography and cross-sectional imaging are typically performed; however, small ovarian tumors may remain occult, particularly in postmenopausal women with atrophic ovaries [6,7]. Consequently, negative imaging findings should not preclude consideration of an ovarian source when clinical suspicion remains high [6,7].

This case also illustrates the limitations of imaging in detecting small androgen-producing ovarian tumors. Computed tomography failed to identify an ovarian mass, and repeated transvaginal ultrasonography could not visualize the ovaries. Definitive diagnosis was achieved only after bilateral salpingo-oophorectomy, which revealed a 0.5-cm Sertoli-Leydig cell tumor in the right ovary and a 0.6-cm pure Leydig cell tumor in the left ovary. The small size of these tumors likely contributed to their absence on preoperative imaging. Similar reports describe androgen-producing ovarian tumors that were difficult to localize preoperatively [5,7]. Therefore, negative imaging does not exclude an ovarian androgen-secreting neoplasm when biochemical findings strongly support an ovarian source [6,7].

The bilateral nature of this case further distinguishes it from previously reported SCSTs, which are overwhelmingly unilateral [1,3]. Furthermore, the presence of two distinct histologic subtypes, a Sertoli-Leydig cell tumor in one ovary and a pure Leydig cell tumor in the contralateral ovary, suggests that these lesions may represent synchronous primary neoplasms rather than metastatic spread from a single primary lesion. Molecular analysis of bilateral DICER1-associated Sertoli-Leydig cell tumors has likewise supported their classification as independent primary neoplasms [8]. Although bilateral androgen-secreting SCSTs have rarely been described [4,5,8], this case highlights the importance of careful pathologic evaluation of both ovaries, even when they appear grossly normal at surgery.

This case emphasizes several important clinical lessons. Persistent or progressive hyperandrogenism in postmenopausal women warrants thorough evaluation, even when imaging studies are unrevealing [6,7]. Biochemical testing can help localize androgen excess and inform subsequent diagnostic evaluation [6,7]. Finally, when clinical suspicion for an occult ovarian androgen-secreting tumor remains high despite negative imaging, surgical management may serve both diagnostic and therapeutic purposes, allowing definitive diagnosis and treatment of otherwise undetectable disease [5-7].

## Conclusions

Despite their rarity, SCSTs should be considered in the differential diagnosis of postmenopausal women presenting with hyperandrogenism, even when imaging is unrevealing. Although these tumors are often benign, malignant potential has been reported and may influence management decisions. This case highlights the diagnostic challenges posed by small, radiologically occult androgen-secreting ovarian tumors. Substantial gaps remain in the understanding of the epidemiology and molecular drivers of these rare tumors.
